# Multidrug-Resistance and Toxic Metal Tolerance of Medically Important Bacteria Isolated from an Aquaculture System

**DOI:** 10.1264/jsme2.ME12049

**Published:** 2012-09-05

**Authors:** Juliana Alves Resende, Vânia L. Silva, Cláudia Oliveira Fontes, Job Alves Souza-Filho, Tamara Lopes Rocha de Oliveira, Cíntia Marques Coelho, Dionéia Evangelista César, Cláudio Galuppo Diniz

**Affiliations:** 1 Department of Parasitology, Microbiology and Immunology, Federal University of Juiz de Fora, Juiz de Fora, Minas Gerais, 36036–631, Brazil; 2 Department of Biology Institute of Biological Sciences, Federal University of Juiz de Fora, Juiz de Fora, Minas Gerais, 36036–631, Brazil

**Keywords:** Drug-resistant bacteria, toxic metal tolerance, aquaculture

## Abstract

The use of antimicrobials and toxic metals should be considered carefully in aquaculture and surrounding environments. We aimed to evaluate medically relevant bacteria in an aquaculture system and their susceptibility to antimicrobials and toxic metals. Selective cultures for enterobacteria (ENT), non-fermenting Gram-negative rods (NFR) and Gram-positive cocci (GPC) were obtained from water samples collected in two different year seasons. The isolated bacteria were biochemically identified and antimicrobial and toxic metal susceptibility patterns were determined. Overall, 407 representative strains were recovered. In general, bacteria isolated from fish ponds showed higher multiple antibiotic resistance indices when compared to those isolated from a water-fed canal. Resistance to penicillin and azithromycin was observed more frequently in the GPC group, whereas resistance to ampicillin and ampicillin/sulbactam or gentamicin was observed more frequently in the ENT and NFR groups, respectively. All the isolated bacteria were tolerant to nickel, zinc, chromium and copper at high levels (≥1,024 μg mL^−1^), whereas tolerance to cadmium and mercury varied among the isolated bacteria (2–1,024 μg mL^−1^). Multidrug-resistant bacteria were more frequent and diverse in fish ponds than in the water-fed canal. A positive correlation was observed between antimicrobial resistance and metal tolerance. The data point out the need for water treatment associated with the aquaculture system.

Considering increasing antimicrobial resistance and its consequences for human and animal medicine, which are widely discussed issues (3), aquaculture activity is particularly relevant due to the enhanced probability of horizontal gene transfer in an aquatic environment between the water microbiota and soil microorganisms (13, 19, 30). Furthermore, the discharge of metals into soil and aquatic ecosystems may favor the selection of antimicrobial-resistant bacteria due to events related to co-selection and cross-resistance (4).

To date, the empirical use of antimicrobials in aquaculture as a profitable commercial activity has been supported by the major concerns underlying fish farming, such as bacteria-associated infectious diseases, which may result in potential risks for the output of fish farming (8). Thus, antibiotic therapy remains important as a prophylactic agent and/or a therapeutic method, especially considering the cost-effective aquaculture production chain (29).

For technical reasons, antimicrobial agents have been introduced as food components or directly into water used for culture (16). Due to the high population densities achieved by the aquatic species during culture conditions, it is difficult to address individual dosages, which results in the introduction of different amounts of various drugs into the environment (5). As a consequence, there is a deposition of antimicrobial residues in sediments, resulting in continuous selective pressure, thereby stimulating the sediment microbiota towards the selection of resistant bacteria (30, 32). In addition, resistance genes are highly persistent and do not disappear from aquaculture sites, even after several years without antibiotic use (34).

Along with the concerns associated with using antimicrobial drugs, toxic metals, often introduced into the environment via anthropogenic or natural sources, may also interfere with different bacterial communities. It is well established that environmental metal contamination has an important role in the maintenance and proliferation of antibiotic-resistant bacteria due to co-selection mechanisms (4). Unlike organic pollutants, chemical and biological agents, toxic metals are not subject to degradation and may remain as agents of selective pressure over long periods (4, 31). Aquaculture systems may come in close contact with toxic metals, since they are directly used as chemical control agents of algae and parasites in fishponds, and fertilizers in the environment, or discharged from industry and sewage (7, 28).

In this regard, identifying microorganisms and their susceptibility patterns to antimicrobial drugs and toxic metals may be useful in determining the persistence of clinically and microbiologically relevant bacteria, and may also help to monitor the potential spread of resistant bacteria by aquaculture activity. This paper focused on the evaluation of the occurrence of medically relevant bacteria, such as enterobacteria (ENT), non-fermenting Gram-negative rods (NFR) and Gram-positive cocci (GPC), and their susceptibility patterns to antimicrobial drugs and toxic metals and the occurrence of plasmids in isolated microorganisms at an experimental Aquaculture Farm in Brazil, which belongs to Minas Gerais Agriculture and Livestock Research Company (EPAMIG).

## Materials and Methods

### Isolation and identification of bacterial samples

Eighty water samples were collected at different sites at the EPAMIG Nile tilapia (*Oreochromis niloticus*, Linnaeus, 1758) Aquaculture Farm in Leopoldina, Brazil, referred to as the water-fed canal (n=8), fish distribution ponds (n=8) and growth ponds (n=64). The samples were collected equally from two different periods: between May and August (2009) (winter/dry season) and from January to March (2010) (summer/rainy season). Water samples (20 mL) were collected from between 0 and 20 cm under the surface, using sterile bottles. All samples were brought to the laboratory and processed within 4 h after collection. They were concentrated to 2 mL by centrifugation at 8000×*g* for 10 min at 4°C.

Selective cultures (0.1 mL) were performed in Hypertonic Manitol and Bile Esculin Agar (Himedia Laboratories, India) for GPC and in Eosin-Methylene Blue Agar (Himedia Laboratories) for ENT and NFR. After incubation (18 to 24 h, 37°C), 1 to 5 different representative colonies were selected and cultivated in Brain–Heart Infusion Agar (Himedia Laboratories) for storage by freezing for further experiments. The GPC were presumptively identified by morphotinctorial characteristics after Gram staining, as well as the ability to hydrolyze esculin and produce catalase. Species identification was performed using the commercial system BBL Crystal Rapid Gram-Positive ID Kit (Becton & Dickinson, USA), according to the manufacturer’s instructions. The Gram-negative bacteria ENT and NFR were presumptively identified by morphotinctorial characteristics after Gram staining, as well as the ability to ferment carbohydrate and be motile. Species identification was performed using the commercial systems Bactray I, II and III (Laborclin, Brazil), according the manufacturer’s instructions.

### Antimicrobial susceptibility assays

The minimum inhibitory concentration (MIC) for antimicrobial drugs and toxic metals were determined by the agar dilution method, according to the Clinical and Laboratory Standard Institute guideline (11). Antibiotic or toxic metals stock solutions were added to melted Mueller-Hinton (HiMedia) agar to obtain final concentrations ranging from 0.06 to 1,024 μg mL^−1^. The antimicrobial drugs were selected on the basis of microbial characteristics and clinical relevance as follows: (i) for GPC, penicillin (MedQuimica, Brazil), oxacillin (MedQuimica), vancomycin (MedQuimica), gentamicin (MedQuimica), azithromycin (Sigma Aldrich, USA), tetracycline (MedQuimica), levofloxacin (MedQuimica), sulphamethoxazole-trimetropim (MedQuimica) and chloramphenicol (MedQuimica); (ii) for ENT, ampicillin (Cellofarm, Brazil), ampicillin-sulbactam (Cellofarm), piperacillin-tazobactam (Novafarma, Brazil), ceftriaxone (Eurofarma, Brazil), cefepime (Biochimico, Brazil), meropenen (Biochimico), gentamicin (Novafarma, Brazil), amikacin (Teuto- Brasileiro Laboratorio, Brazil), levofloxacin, sulphamethoxazole-trimetropim and chloramphenicol; and (iii) for NFR, piperacillin-tazobactam, ceftazidime (Eurofarma, Brazil), cefepime, gentamicin, amikacin, sulphamethoxazole-trimetropim and chloramphenicol. The reference strains *Enterococcus faecalis* ATCC 51299, *Staphylococcus aureus* ATCC 29213 and *Escherichia coli* ATCC 25922 were included as controls in the antimicrobial susceptibility assays for Gram-positive or Gram-negative bacteria and all tests were performed in duplicate. Using CLSI guidelines, the isolates were classified as sensitive, intermediate, or resistant to the tested antimicrobial agents (11). To determine the level of antibiotic resistance of the individual isolated bacteria, the multiple antibiotic resistance (MAR) index was calculated according to the literature (18), by dividing the number of antibiotics to which the isolate was resistant by the total number of antibiotics to which the isolates were exposed. A MAR value >0.2 was indicative of multiple antibiotic-resistant bacteria.

The toxic metals were selected on the basis of their environmental availability and biologic relevance: nickel (NiCl_2_·6H_2_O), zinc (ZnSO_4_·7H_2_O), mercury (HgCl_2_), cadmium (CdCl_2_·H_2_O), chromium (Cr(NO_3_) _3_) and copper (CuSO_4_) (Vetec, Brazil). The reference strains *E. faecalis* ATCC 51299, *S. aureus* ATCC 29213 and *Escherichia coli* ATCC 25922 were used for quality control. All tests were performed in duplicate. The MIC was determined as the lowest concentration of metal salt that completely inhibited bacterial growth. The results were expressed as the level of tolerance based on toxic concentrations of these substances to other biological systems and compared to the reference values of MIC for the strain *E. coli* K-12, according to the literature (2, 22).

### Statistical analysis

One-way analysis of variance (ANOVA) was applied to compare the distribution of different bacterial groups in different water sources (water-fed canal, fish distribution ponds and growth ponds) both in dry (winter) and rainy seasons (summer). The significance level was set as p<0.05. The analysis of the association between bacterial strains with MAR index ≥0.2 and high tolerance to toxic metals was assessed by calculating the odds ratio (OR) (6). The confidence interval used was 95%. An OR value of ≤1.0 indicated a negative correlation, *i.e.*, the probability is lower in the first group than in the second or the condition under study is equally likely in both groups. An OR value >1.0 indicated a positive correlation.

## Results

Four hundred and seven (n=407) bacterial samples were isolated. Representative strains of ENT were the most frequent (n=195, 47.91%), followed by NFR (n=115, 28.25%) and GPC (n=100, 23.84%). According to ANOVA, no significant difference was observed among the sampled sites considering the variations in the same bacterial group (p=0.873). However, considering the frequency of microbial recovery in different water sources (water-fed canal, fish distribution ponds and growth ponds), ENT strains were the most abundant in every environment, both in dry and rainy periods and especially in the fish distribution ponds (p=0.01), whereas NFR and GPC were regularly distributed.

Microbial identification is shown in Table 1. Among the GPC, 29 different species were identified, although 6 strains (6.19%) had no identification score according to the methodology used in this study. The most prevalent GPC species were *Enterococcus faecalis* (17.53%), *Staphylococcus epidermidis* (17.53%), *Enterococcus avium* (6.19%) and *Micrococcus luteus* (6.19%). Out of the 310 Gram-negatives rods, 62.9% were ENT, while 37.10% were NFR. Among the ENT, the most prevalent species were *Klebsiella* sp. (16.93%), *Serratia* sp. (16.91%), *Hafnia* sp. (13.3%), *Escherichia* sp. (10.26%), *Enterobacter* sp. (7.7%) and *Citrobacter* sp. (6.66%). Among the NFR, the most prevalent species were *Pseudomonas* sp. (29.58%), *Burkholderia* sp. (20.84%), *Ochrobactrum* sp. (14.78%) and *Chromobacterium* sp. (8.7%). As observed with GPC, 6 NFR strains (6.09%) did not have an identification score according to the methodology used in this study.

The antimicrobial drug susceptibility patterns are shown in Table 2. Considering the GPC, antimicrobial resistance levels higher than 10.0% were observed against penicillin G, oxacillin, gentamicin, azithromycin and tetracycline. Azithromycin was the least effective drug and resistance rates of 48% were observed. In contrast, levofloxacin, sulphamethoxazole-trimetropim and vancomycin were the most effective antimicrobials, with sensitivity rates of 91, 93 and 96.5%, respectively.

Overall, for ENT strains, antimicrobial resistance was observed against all the tested drugs, with resistance rates higher than 10.0% observed against ampicillin, ampicillin-sulbactam and ceftriaxone, whereas the most effective antimicrobials were piperacillin-tazobactam (4.85%), amikacin (3.8%) and levofloxacin (0.55%). In an unusual pattern, 8.10% of the ENT strains were resistant to meropenem and were identified as follows: *Serratia odorifera* (n=3), *S. liquefaciens* (n=1), *Klebsiella pneumoniae* (n=3), *K. ozaenae* (n=2), *Escherichia coli* (n=2), *Enterobacter cancerogenus* (n=2) and *Hafnia alvei* (n=3).

Finally, for NFR bacteria, antimicrobial resistance was also observed against all the tested drugs, with rates higher than 30.0% for gentamicin, chloramphenicol and amikacin. Resistance rates lower than 30% were observed against piperacillin-tazobactam, ceftazidime, cefepime and sulfamethoxazole-trimethoprim. The last was the most effective drug with a microbial sensitivity rate of 88.6%.

The multiple antibiotic resistance (MAR) index for resistant bacteria is presented in Table 3. According to this parameter, bacteria isolated from the water-fed canal were shown, in general, to be less multiple antibiotic-resistant than the same microbial groups isolated from the distribution and growth ponds. Of the GPC isolated from the water-fed canal, 40% were resistant to at least one drug and 10% of these resistant strains showed MAR >0.2. Considering the fish distribution and growth ponds, the frequency of resistance was 67.5% and 55.8% displayed MAR >0.2. The same was observed for ENT and NFR isolated from the water-fed canal in which 55.8% (ENT) and 70% (NFR) were resistant to at least one drug and 5.9% and 40% had a MAR >0.2. In addition, frequencies of resistance of 73.2% and 78.6% were observed for ENT and NFR, respectively, isolated from distribution and growth ponds, with MAR >0.2 recorded for 21.4% of resistant ENT and 59.2% of resistant NFR.

Susceptibility to toxic metals for the isolated micro-organisms is shown in Table 4, and is presented in terms of MIC_50_, MIC_90_ and the range of MICs. As there are no standards for toxic metal susceptibility, and the tests were performed according to the CLSI guidelines for antimicrobial drugs (CLSI 2010), the results were referred to as high or low tolerance to toxic metals.

Cadmium tolerance was higher for ENT and NFR bacteria (MIC >100 μg mL^−1^ observed for 80.3 and 71.7%, respectively) when compared to GPC strains, for which MIC <100 μg mL^−1^ was observed for 84.8% (15.2% of tolerant strains). High tolerance to mercury (MIC >12.5 μg mL^−1^) was observed for all bacterial groups at a high frequency: 92.4% for GPC, 78.8% for ENT and 66.4% for NFR. A high frequency (>97%) of bacteria tolerant to chromium, nickel, zinc and copper was observed in all evaluated groups. Overall, MIC_50_ for these toxic metals was at least 1,024 μg mL^−1^.

Besides the toxic metal-tolerant bacteria isolated from all different water sources (water-fed canal, fish distribution ponds and growth ponds), both positive and negative correlations were observed between bacteria with MAR >0.2 and multi-metal tolerances (Table 5). OR values considering antimicrobial-resistant bacteria and tolerance to nickel, zinc, chromium and copper were equal to 1.0 and indicated no correlation between multidrug resistance and high tolerance to these toxic metals; however, considering mercury, the OR values for all antimicrobial-resistant microorganisms were >1.0, indicating the possibility of a positive correlation between bacteria with MAR ≥0.2 and metal tolerance. Regarding cadmium tolerance, no correlation was detected among antimicrobial-resistant ENT (OR<1.0), although a positive correlation was observed for GPC and NFR (OR>1.0).

## Discussion

The occurrence of bacteria strains such as coagulase-negative staphylococci, potential and opportunistic representatives of enterobacteria and highly opportunistic NFR such as *Pseudomonas* sp. and *Burkholderia* sp. justifies the microbiological relevance of correctly handling aquaculture activity. Taken together, all these detected bacterial groups confirmed the persistence of putative microorganisms in the aquatic environment, which have potential implications for public health (16, 37, 38).

According to the literature, GPC was expected to be the least prevalent and the most accepted explanation was that these bacteria are not frequent in the aquatic environment; however, its detection has been shown to be related to external interference by humans and other animals through environmental contamination (9).

Although aquaculture is a growing commercial activity in Brazil, only a few studies have focused on microbiological quality and the risks associated with the aquatic environment. Regarding the ENT and NFR groups in particular, their recovery at higher rates from distribution and fish ponds, compared to GPC, shows that the system evaluated has microbial diversity similar to that observed by other authors in different geographical regions, including Brazil (9, 26) and other regions in South America such as Chile (23), southeast Asia (17) and Australia (1), with a similar climate.

The GPC were highly resistant to azithromycin, tetracycline and oxacillin. It is well known that several genetic markers linked to these phenotypes are located in mobile DNA molecules; however, some bacterial species such as enterococci and pediococci are intrinsically resistant to several drugs (10, 25). In fact, the fish ponds yielding the bacterial biomass may contribute to the spread of such genetic markers through horizontal transfer (bacterial recombination) as selective pressure is imposed. The release of such microorganisms may affect the surrounding environments and consumer safety (13).

High resistance rates against most of the antimicrobial drugs tested, especially ampicillin, were observed for ENT strains. The dynamics of beta-lactam resistance in this study, considering the association of beta-lactam and betalactamase inhibitor (ampicillin-sulbactam and piperacillin-tazobactam) with cephalosporins, suggest that several of the resistant strains might be beta-lactamase producers. From clinical and epidemiological perspectives, this is extremely relevant and may represent both the spread and the selection of antimicrobial-resistant bacteria by aquaculture activity. Other authors have also documented the occurrence of bacteria resistant to beta-lactams in aquatic environments (14, 36).

As meropenem remains an effective drug against most of the Enterobacteriaceae, it is well accepted that carbapenemase activity among these bacteria occurs as a result of ESBL, AmpC and metallo-β-lactamase activity (11). Although some of these enzymes may be chromosomally encoded in several Gram-negative bacteria, such as anaerobic and glucose non-fermenting rods, it is well documented that in enterobacteria, especially *Serratia*, *Klebsiella*, *Enterobacter*, *Morganella*, *Proteus* and some *E. coli* strains, carbapenemases may be carried also on plasmids (12, 20, 21, 35). Thus, it is of great concern and such resistant phenotypes require monitoring, especially in bacteria isolated from environmental sources.

Regarding NFR, high rates of antimicrobial resistance were observed against almost all the antimicrobials tested. It is accepted that NFR are among the most drug-resistant bacteria and active monitoring of this phenomenon is very important, with consequences for public health (3). Furthermore, according to the literature, resistance to gentamicin and other antimicrobials is highly prevalent in aquatic environments (15).

Overall, the high frequency of MAR >0.2 among the bacteria isolated in the distribution and fish ponds indicates aquaculture activity as a possible high risk of spreading resistance genes. The abundance of these multidrug-resistant bacteria may reflect a microbial adaptive response to the empirical use of antimicrobials as prophylactics or therapeutics in fish farming (27, 30).

Different studies have reported toxic metal tolerance among bacteria isolated from aquatic environments (22, 24); however, there is a lack of technical standards in these experimental designs, resulting in difficulties for data comparison. To avoid this lack of technical standards, experiments for testing toxic metal susceptibility patterns among the isolated bacteria were performed by the agar dilution technique, as recommended for antimicrobial susceptibility testing by the Clinical Laboratory Standards Institute (11).

According to the literature, both Gram-positive and Gram-negative bacteria are able to resist toxic metals, which are widely spread in the natural environment. Thus, it is not surprising that toxic metal resistance genes are commonly found in environmental bacteria and these genes may confer co-resistance or cross-resistance to antimicrobial drugs (4, 22). These co-selection mechanisms include co-resistance, *i.e.*, different resistance determinants present on the same genetic element such as a plasmid, and cross-resistance, *i.e.*, a single phenotype may be associated with both antimicrobial and metal resistance, such as an active efflux pump (4). In this way, the higher rates of toxic metal tolerance at high levels detected in most of the bacterial strains isolated in this study may be the result of toxic metal contamination with fertilizers in the environment, since the aquaculture farm evaluated is in agricultural and livestock areas. Moreover, it could be a consequence of the use of some toxic metals, such as copper, in the chemical control of algae and parasites in fishponds (28). Cadmium is widely used in industry and as pesticides and fertilizers (7); however, the use of mercury-based fungicide in the paper industry, agriculture and hospital disinfectants may be linked to selective pressure imposed on the open environment (33).

Taken together, the results confirm that aquaculture as currently practiced may have important public health consequences due to the occurrence of antimicrobial resistance in pathogenic and other types of bacteria in various environments. Although antibiotic therapy remains important as a prophylactic agent and/or therapeutic method in aquaculture, the isolated bacteria were highly resistant to antimicrobials used in human and animal medicine. The positive correlation between multidrug resistance and high tolerance to toxic metals is of special concern, since these substances are used in aquaculture and are commonly discharged into aquatic environments.

Further prospective studies are needed to better assess microbial diversity and sanitary risks in the aquaculture environment, since this activity is increasingly becoming one of the most profitable commercial activities in both developed and developing geographical regions around the world. In-depth discussions are needed concerning the use of antimicrobial drugs and toxic metals in aquaculture and surrounding environments. Other methodological approaches are required to better address the association of multidrug resistance or toxic metal tolerance with the occurrence of plasmids in the aquatic environment.

## Figures and Tables

**Table 1:** Species distribution of bacterial strains isolated from different water sources showing the frequency of identification among Gram-positive cocci (GPC), enterobacteria (ENT) and non-fermenting Gram-negative rods (NFR)

Bacterial group (n) and frequency of species identification (%)		

GPC (n=97)	ENT (n=195)	NFR (n=115)
*Enterococcus faecalis* (17.53)	*Hafnia alvei* (13.3)	*Burkholderia cepacia* (16.49)
*Staphylococcus epidermidis* (17.53)	*Klebsiella ozaenae* (10.26)	*Ochrobactrum anthropi* (14.78)
*Enterococcus avium* (6.19)	*Escherichia coli* (8.21)	*Pseudomonas pseudoalcaligenes* (9.57)
*Micrococcus luteus* (6.19)	*Serratia odorifera* (7.69)	*Chromobacterium violaceum* (8.70)
*Enterococcus casseliflavus/gallinarum* (4.12)	*Plesiomonas shigelloides* (6.67)	*Achromobacter denitrificans* (7.83)
*Staphylococcus auricularis* (4.12)	*Serratia liquefaciens* (4.10)	*Stenotrophomonas maltophilia* (7.83)
*Enterococcus faecium* (3.09)	*Cedecea davisae* (3.59)	*Pseudomonas fluorescens* (6.09)
*Pediococcus pentosaceus* (3.09)	*Citrobacter freundii* (3.59)	*Pseudomonas aeruginosa* (5.22)
*Staphylococcus gallinarum* (3.09)	*Enterobacter cancerogenus* (3.59)	*Pseudomonas stutzeri* (2.61)
*Staphylococcus saprophyticus* (3.09)	*Klebsiella oxytoca* (3.08)	*Burkholderia pseudomallei* (2.61)
*Staphylococcus warneri* (3.09)	*Klebsiella pneumoniae* (3.08)	*Pseudomonas putida* (1.74)
*Staphylococcus xylosus* (3.09)	*Citrobacter amalonaticus* (2.56)	*Burkholderia pickettii* (1.74)
*Gemella morbillorum* (2.06)	*Salmonella choleraesuis* (2.56)	*Pseudomonas alcaligenes* (0.87)
*Staphylococcus kloosii* (2.06)	*Serratia plymuthica* (2.56)	*Pseudomonas mendocina* (0.87)
*Aerococcus urinae* (1.03)	*Tatumella ptyseos* (2.56)	*Pseudomonas orizyhabitans* (0.87)
*Alloiococcus otitidis* (1.03)	*Edwardsiella tarda* (2.05)	*Pseudomonas pseudomallei* (0.87)
*Enterococcus durans* (1.03)	*Enterobacter sakazakii* (2.05)	*Pseudomonas* sp. (0.87)
*Enterococcus raffinosus* (1.03)	*Escherichia fergusonii* (2.05)	*Alcaligenes piechaudii* (0.87)
*Helcococcus kunzii* (1.03)	*Shigella dysenteriae* (2.05)	*Bergeyella zoohelcum* (0.87)
*Kytococcus sedentarius* (1.03)	*Shigella flexneri* (2.05)	*Bordetella bronchiseptica* (0.87)
*Lactococcus lactis* (1.03)	*Cedecea lapagei* (1.54)	*Brevundimonas vesiculares* (0.87)
*Leuconostoc citreum* (1.03)	*Enterobacter asburiae* (1.03)	*Sphingomonas paucimobilis* (0.87)
*Staphylococcus capitis* (1.03)	*Enterobacter gergoviae* (1.03)	Non-identified strains (6.09)
*Staphylococcus intermedius* (1.03)	*Kluyvera ascorbata* (1.03)	
*Staphylococcus pasteuri* (1.03)	*Pantoea dispersa* (1.03)	
*Staphylococcus simulans* (1.03)	*Providencia stuartii* (1.03)	
*Staphylococcus warneri* (1.03)	*Serratia marcescens* (1.03)	
*Streptococcus gordonii* (1.03)	*Citrobacter koseri* (0.51)	
*Streptococcus mutans* (1.03)	*Klebsiella ornithinolytica* (0.51)	
Non-identified strains (6.19)	*Kluyvera cryocrescens* (0.51)	
	*Rahnella aquatilis* (0.51)	
	*Salmonella enteritidis* (0.51)	
	*Serratia ficaria* (0.51)	
	*Serratia fonticola* (0.51)	
	*Serratia* sp. (0.51)	
	*Shigella sonnei* (0.51)	

**Table 2:** Drug susceptibility patterns of bacteria recovered from a water-fed canal, distribution pond and fish ponds in the EPAMIG Aquaculture Farm

Microbial group and antimicrobial drugs		Minimum inhibitory concentrations (μg/mL^−1^)			Susceptibility patterns (%)^a^		

		MIC_50%_	MIC_90%_	Range	S	IR	R
CGP^b^	Penicillin G	0.50	8.00	0.06–32.00	55.20	—	44.80
	Oxacillin	0.50	2.00	0.06–64.00	78.10	—	21.80
	Vancomycin	1.00	2.00	0.06–16.00	96.50	3.50	—
	Gentamicin	2.00	512.00	0.12–512.00	86.00	—	14.00
	Azithromycin	4.00	64.00	0.06–128.00	52.00	6.00	42.00
	Tetracycline	0.50	256.00	0.06–256.00	70.00	8.00	22.00
	Levofloxacin	0.50	2.00	0.06–4.00	91.00	1.00	8.00
	Sulphamethoxazole-trimetropim	1.20	9.50	0.30–152.00	93.00	—	7.00
	Chloramfenicol	4.00	8.00	0.50–64.00	89.00	1.00	10.00

ENT^c^	Ampicillin	32.00	512.00	0.06–>024.00	23.80	9.72	66.40
	Ampicillin-sulbactam	16.00	128.00	0.06–512.00	44.40	16.20	39.40
	Piperacillin-tazobactam	2.00	16.00	0.06–512.00	90.80	4.35	4.85
	Ceftriaxone	0.25	16.00	0.06–1,024.00	80.00	2.70	17.30
	Cefepime	0.06	4.00	0.06–512.00	90.30	—	9.70
	Meropenen	0.12	4.00	0.06–256.00	90.80	1.10	8.10
	Gentamicin	2.00	8.00	0.06–>1,024.00	86.50	4.32	9.18
	Amikacin	2.00	8.00	0.12–>1,024.00	93.50	2.70	3.80
	Levofloxacin	0.06	1.00	0.06–8.00	99.45	—	0.55
	Sulphamethoxazole-trimetropim	4.80	19.00	0.30–1,216.00	93.50	—	6.50
	Chloramfenicol	4.00	16.00	0.50–256.00	81.65	11.35	7.00

NFR^d^	Piperacillin-tazobactam	8.00	256.00	0.06–1,024.00	58.65	15.85	25.50
	Ceftazidime	8.00	256.00	0.06–>1,024.00	70.00	2.70	27.30
	Cefepime	2.00	128.00	0.06–1,024.00	72.72	5.28	22.00
	Gentamicin	32.00	256.00	0.06–>1,024.00	44.55	2.65	52.80
	Amikacin	8.00	128.00	0.25–512.00	60.40	7.05	32.50
	Sulphamethoxazole-trimetropim	4.80	76.00	0.30–608.00	88.60	—	11.40
	Chloramfenicol	16.00	64.00	0.50–128.00	41.05	24.65	34.30

a S: sensitivity; IR: intermediate resistance; R: resistance;

b GPC: Gram-positive cocci (n=97);

c ENT: Gram-negative rods from the *Enterobacteriaceae* family (n=195);

d NFR: non-fermenting Gram-negative rods (n=115).

**Table 3:** Frequency of drug-resistant bacteria and multiple antibiotic resistance (MAR) index among microbial groups isolated from a water-fed canal, distribution pond and fish ponds in the EPAMIG Aquaculture Farm

Water samples	Microbial group	Frequency of resistance (%)	MAR (frequency of determination %)		

			<0.2	>0.2	Range
Feed water canal	GPC^a^	40.00	30.00	10.00	0.11–0.22
	ENT^b^	58.80	52.90	5.90	0.09–0.45
	NFR^c^	70.00	30.00	40.00	0.14–0.48

Distribution and growth ponds	GPC	67.50	11.70	55.80	0.11–0.66
	ENT	73.20	51.80	21.40	0.09–0.81
	NFR	78.60	19.40	59.20	0.14–0.71

a GPC: Gram-positive cocci;

b ENT: Gram-negative rods from the *Enterobacteriaceae* family;

c NFR: non-fermenting Gram-negative rods.

**Table 4:** Toxic metal susceptibility patterns of bacteria recovered from a water-fed canal, distribution pond and fish ponds in the EPAMIG Aquaculture Farm

Microorganism and toxic metals		Minimum inhibitory concentrations (μg/mL^−1^)			Frequency of tolerant strains (%)^d^

		MIC_50%_	MIC_90%_	Range	
CGP^a^	Nickel (NiCl_2_·6H_2_O)	>1,024.00	>1,024.00	512.00–>1,024.00	100.00
	Zinc (ZnSO_4_·7H_2_O)	1,024.00	>1,024.00	64.00–>1,024.00	100.00
	Mercury (HgCl_2_)	16.00	32.00	4.00–256.00	92.40
	Cadmium (CdCl_2_·H_2_O)	32.00	128.00	2.00–512.00	15.20
	Chromium (Cr(NO_3_)_3_)	>1,024.00	>1,024.00	256.00–>1,024.00	97.80
	Copper (CuSO_4_)	1,024.00	1,024.00	128.00–1,024.00	98.90

ENT^b^	Nickel (NiCl_2_·6H_2_O)	>1,024.00	>1,024.00	512.00–>1,024.00	100.00
	Zinc (ZnSO_4_·7H_2_O)	>1,024.00	>1,024.00	510.00–>1,024.00	100.00
	Mercury (HgCl_2_)	16.00	32.00	4.00–256.00	78.80
	Cadmium (CdCl_2_·H_2_O)	256.00	1,024.00	8.00–>1024.00	80.30
	Chromium (Cr(NO_3_)_3_)	>1,024.00	>1,024.00	1,024.00–>1,024.00	100.00
	Copper (CuSO_4_)	1,024.00	>1,024.00	256.00–>1,024.00	100.00

NFR^c^	Nickel (NiCl_2_·6H_2_O)	>1,024.00	>1,024.00	512.00–>1,024.00	100.00
	Zinc (ZnSO_4_·7H_2_O)	>1,024.00	>1,024.00	256.00–>1,024.00	100.00
	Mercury (HgCl_2_)	16.00	32.00	4.00–128.00	66.40
	Cadmium (CdCl_2_·H_2_O)	128.00	1,024.00	8.00–>1,024.00	71.70
	Chromium (Cr(NO_3_)_3_)	>1,024.00	>1,024.00	256.00–>1,024.00	99.10
	Copper (CuSO_4_)	1,024.00	1,024.00	256.00–>1,024.00	100.00

a GPC: Gram-positive cocci (n=97);

b ENT: Gram-negative rods from the *Enterobacteriaceae* family (n=195);

c NFR: non-fermenting Gram-negative rods (n=115);

d The break point values for high metal tolerance were: 200 μg mL^−1^ for nickel, 200 μg mL^−1^ for zinc, 12.5 μg mL^−1^ for mercury, 100 μg mL^−1^ for cadmium, 800 μg mL^−1^ for chromium, 200 μg mL^−1^ for copper (2, 26).

**Table 5:** Correlation between multidrug-resistant bacteria (MAR ≥0.2) recovered from a water-fed canal, distribution and fish ponds in the EPAMIG Aquaculture Farm and high tolerance to toxic metals

Toxic metal	Odds ratio^a^ (95% confidence interval)		

	GPC^b^	ENT^c^	NFR^d^
Nickel (NiCl_2_·6H_2_O)	1.00 (0.50–1.80)	1.00 (0.60–1.60)	1.00 (0.50–1.60)
Zinc (ZnSO_4_·7H_2_O)	1.20 (0.10–19.40)	1.00 (0.60–1.60)	1.00 (0.50–1.60)
Mercury (HgCl_2_)	3.20 (0.50–17.50)	1.10 (0.40–2.60)	3.50 (1.50–8.00)
Cadmium (CdCl_2_·H_2_O)	6.10 (1.20–29.40)	0.50 (0.20–1.10)	5.80 (2.40–14.10)
Chromium (Cr(NO_3_)_3_)	1.20 (0.10–19.40)	1.00 (0.60–1.60)	1.00 (0.50–1.60)
Copper (CuSO_4_)	1.00 (0.50–1.80)	1.00 (0.60–1.60)	1.00 (0.50–1.60)

a OR=1: absence of correlation; OR>1: positive correlation; OR<1: negative correlation;

b GPC: Gram-positive cocci;

c ENT: Gram-negative rods from the *Enterobacteriaceae* family;

d NFR: non-fermenting Gram-negative rods.
